# The potential of mesenchymal stem cells in the management of radiation enteropathy

**DOI:** 10.1038/cddis.2015.189

**Published:** 2015-08-06

**Authors:** P-Y Chang, Y-Q Qu, J Wang, L-H Dong

**Affiliations:** 1 Department of Radiation Oncology, The First Bethune Hospital of Jilin University, Changchun 130021, China; 2 Electrochemical State Key Laboratory, Changchun Institute of Applied Chemistry Academy of Science, Changchun 130021, China

## Abstract

Although radiotherapy is effective in managing abdominal and pelvic malignant tumors, radiation enteropathy is still unavoidable. This disease severely affects the quality of life of cancer patients due to some refractory lesions, such as intestinal ischemia, mucositis, ulcer, necrosis or even perforation. Current drugs or prevailing therapies are committed to alleviating the symptoms induced by above lesions. But the efficacies achieved by these interventions are still not satisfactory, because the milieus for tissue regeneration are not distinctly improved. In recent years, regenerative therapy for radiation enteropathy by using mesenchymal stem cells is of public interests. Relevant results of preclinical and clinical studies suggest that this regenerative therapy will become an attractive tool in managing radiation enteropathy, because mesenchymal stem cells exhibit their pro-regenerative potentials for healing the injuries in both epithelium and endothelium, minimizing inflammation and protecting irradiated intestine against fibrogenesis through activating intrinsic repair actions. In spite of these encouraging results, whether mesenchymal stem cells promote tumor growth is still an issue of debate. On this basis, we will discuss the advances in anticancer therapy by using mesenchymal stem cells in this review after analyzing the pathogenesis of radiation enteropathy, introducing the advances in managing radiation enteropathy using regenerative therapy and exploring the putative actions by which mesenchymal stem cells repair intestinal injuries. At last, insights gained from the potential risks of mesenchymal stem cell-based therapy for radiation enteropathy patients may provide clinicians with an improved awareness in carrying out their studies.

## Facts

Radiation enteropathy severely affected the quality of life of cancer patients nowadays.
Preclinical data suggest the pro-regenerative effects of mesenchymal stem cells on irradiated intestine.
Epinal case report reveals the specific effectiveness of mesenchymal stem cells in managing pelvic radiotherapy-induced lesions in rectum and bladder lesions.

## Open Questions

Due to most of radiation enteropathy patients are cancer survivors, is really that mesenchymal stem cells will initiate or promote their tumor growth?
How to carry out a clinical trial for evaluating the therapeutic potentials of mesenchymal stem cells for radiation enteropathy?
Will the mesenchymal stem cell-based therapy be an attractive tool for clinicians in managing radiation enteropathy patients in the future?

Radiotherapy is powerful in treating malignant tumors. According to the published data, at least 50% of cancer patients need radiotherapy during their treatment course, and approximately 25% of solid tumors undergo complete remission after radiotherapy.^1^ However, damage to healthy tissue within the radiation field remains unavoidable. For abdominopelvic radiotherapy, the intestine is defined as an organ at risk (OAR). Herein, small intestine commonly presents acute injuries due to its high *α*/*β* ratio of >10 Gy according to linear-quadratic (L-Q) model. Besides, the estimated *α*/*β* ratio in rectum varies between 4.8 Gy and 5.4 Gy, commonly allowing for grade ≥2 toxicity happening.^2, 3^ Radiation-induced intestinal injuries/toxicities are known as radiation enteropathy (RE), which can be classified into two phases. Early RE commonly occurs within 3 months of radiotherapy, with an incidence of ~50%.^4^ Late RE can be observed from 1 to 20 years post radiotherapy, with the incidence of 2–20%.^5, 6^ Several factors are involved in the development of late RE, including progressive cell loss and vascular obliteration in irradiated intestine, which will result in emergent or even fatal complications, such as obstruction, perforation, intestinal necrosis or acute hemorrhage.^6, 7^

Current clinical interventions for early RE mainly aim to relieve abdominal pain and diarrhea through spasmolysis and anti-edema drugs, maintaining electrolyte balance through conditional nutrient supplementation and alleviating inflammation or infection using antioxidants, glucocorticoids or antibiotics.^8^ For late RE, lesioned intestine can be managed merely by surgery.^8^ However, resection of diseased intestine appears to be not very effective, because the fibrogenesis in irradiated intestine could not be inhibited. Additionally, intestinal adhesion following surgery and dystrophia induced by removing a large portion of intestine adversely affect patient quality of life.^9^ In recent years, the outcome from clinical studies exhibited the effectiveness of Pentoxifylline-Vitamin E in preventing intestinal fibrosis.^10, 11^ Meanwhile, several preclinical studies proposed some available agents for managing late RE, including ROCK inhibitor (Y-27632),^12^ Pravastatin^13^ and Simvastatin.^14^ In addition to developing potential drugs, several preclinical studies were carried out for evaluating the therapeutic potentials of mesenchymal stem cells (MSCs) for RE.

MSCs, a population of undifferentiated cells deriving from early ectoderm and can be harvested from various tissues and organs.^15^ MSCs can secret various types of growth factors, immune mediators and anti-fibrotic effectors, which are potent in mediating tissue regeneration.^16, 17, 18^ And several clinical trials revealed the immunomodulatory benefits of MSCs in treating graft *versus* host disease (GVHD), inflammatory bowel disease (IBD), systemic lupus erythematosus (SLE) and arthritis.^19, 20, 21, 22^ Moreover, four patients, suffering from pelvic radiotherapy-induced injuries in rectum and in bladder, were successfully treated in Epinal Medical Center by using MSCs.^6, 23^ The effectiveness of MSCs lies in reducing abdominal pain, stanching rectal hemorrhage and healing fistula.^23^ On this basis, we propose that managing RE patients by using MSCs will be an attractive therapeutic approach in the future.

In this review, we will build on evidence of an effect of MSCs on irradiated intestine by discussing the actions behind this effect, together with evidence relating to advances in anticancer therapy by using MSCs, to suggest the feasibility of MSCs in treating cancer patients with RE. At last, we will introduce the future efforts into carrying out MSC-related clinical trials for managing RE.

## Potential Factors Involved in Pathogenesis of RE

Refractory lesions in the irradiated intestine develop through a lengthy process comprising multiple steps and contributory factors, although the mechanisms involving in RE pathogenesis remain unclear. Radiation-induced injuries refer to the histopathological changes in the epithelium and endothelium, where the oxidative stress and extended inflammation played a predominant role in leading to lesions, including ischemia, ulcer, necrosis or fibrosis.^24, 25^ In this section, current understanding on the pathogenesis of RE will be shared.

### Epithelial event: Intestinal stem cell injury-induced de-epithelialization

**Figure 1:**
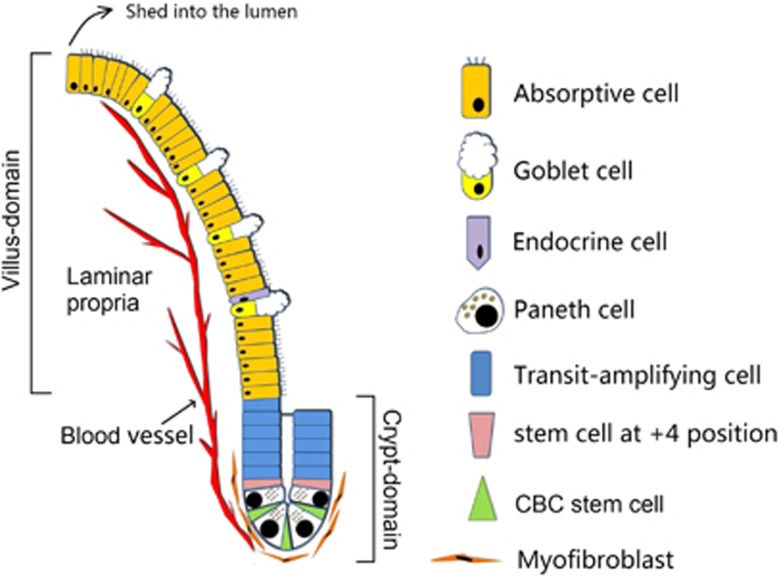
The structure of villus-crypt axis. This figure shows the homeostasis of intestinal epithelium regulating by CBC stem cells

The intestinal epithelium has a rapid self-renewal capacity, with a complete turnover time of ~96 h due to rapid cycling of cells within the crypts of Liberkühn.^26^ The crypt base columnar (CBC) stem cells are responsible for maintaining homeostasis of intestinal epithelium by their producing progeny: transit-amplifying cells, which are committed into mature epithelial cells after 4–5 divisions (Figure 1).^27, 28^

The CBC stem cells are radiosensitive.^29^ It was reported that irradiation doses of at least 0.01 Gy are sufficient to result in apoptosis in 10% of CBC stem cells.^30^ The pro-apoptotic effects of ionizing irradiation on CBC stem cells are dependent on their cell-cycle stage.^29^ Ionizing irradiation will cause more DNA double-strand breaks (DSB) in G2/M phase than in G1/S phase.^31^ The average dividing time of CBC cells is reported to be 21.5 h, which classifies them as rapid cycling and radiosensitive cells thereby.^32^

**Figure 2:**
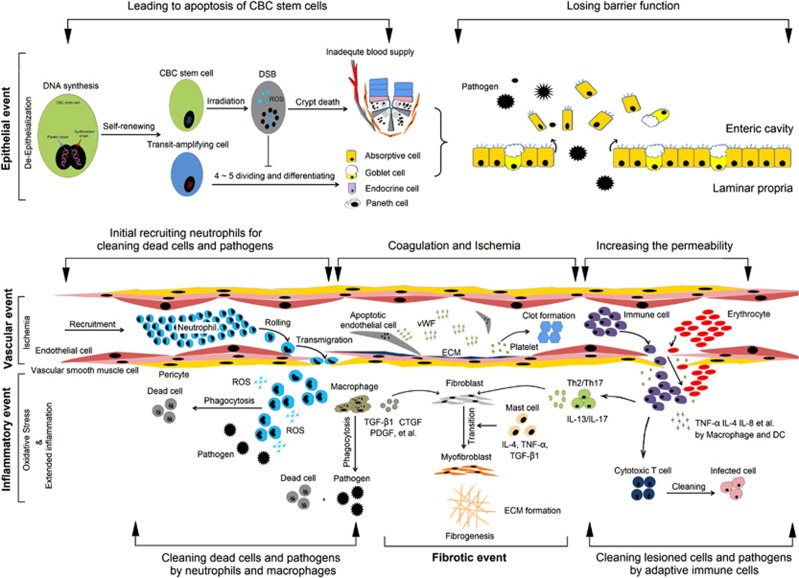
The pathogenesis of radiation enteropathy. Four events are involved in the pathogenesis of radiation enteropathy, including de-epithelization, ischemia, oxidative stress and fibrogenesis

Recent evidence suggested that the CBC stem cells are indispensable for epithelial regeneration upon being irradiated.^33^ If the intestine receives the doses between 6 Gy and 12 Gy, CBC stem cells can be replenished by another pool of intestinal stem cells at the 4+ position of crypts.^34, 35^ Yet, if the irradiation doses are higher than 12 Gy, the CBC stem cells will rapidly die along with subsequent depletion of Paneth cells, who form the niches of CBC stem cells.^33, 36^ Together with the following processes, such as vascular damage-induced ischemia, oxidative stress and extended inflammation in irradiated sites, the milieus feeding CBC stem cells are further deteriorated, which even results in crypt death.^37^ Ultimately, the barrier function of epithelium is lost thereby (Figure 2).

### Vascular event: endothelial injury-induced ischemia

The endothelial cells are primary targets of ionizing irradiation, and the apoptosis of endothelial cells accounts for the severity of lesions within irradiated intestine.^38, 39^ Evidence lie in that during the first 4 h after lethal irradiation, the apoptotic cells, most of which are positive for CD31, are mainly located in the laminar propria of the villi rather than the epithelial layer.^40^ However, at 10 h post irradiation, numerous apoptotic cells are diffusely located in the epithelial layer from villi to crypt compartments rather than the laminar propira.^40^ This switch of apoptosis from endothelial cells to epithelial cells indicates that injuries in irradiated intestinal cells are first occurred in endothelium.^39, 40^

After endothelial injury occurring, sub-endothelial extracellular matrix (ECM) components are exposed to platelets, which initiate hemostasis mechanisms by forming clots.^41^ The coagulation cascade is hyperactivated by excessive secretion of von Willebrand factor (vWF) from injured endothelial cells, which results in vascular occlusion ultimately.^42^ Then, vascular permeability will be increased, which leads to hyperemia or hemorrhage within injured sites.^43^ As a result, the irradiated intestine has a poor blood supply (Figure 2).

### Inflammatory event: increasing oxidative stress and extending inflammation

The chemical reactions that reduce cell viability in irradiated tissues have been identified as excessive production of ROS, such as superoxide radicals and hydrogen peroxide, which derive from intracellular water oxidized by radiation energy and from mitochondria.^44, 45^ Under this condition, ROS can activate the signaling pathways leading to cell death in irradiated sites.^46, 47^ Following these processes, leucocytes roll to the damaged intestine in a short time for eliminating dead cells (Figure 2).^48, 49^

Regarding the development of inflammation, current opinions believe that the molecular reactions in this process occur at sites extremely close to the vascular endothelium.^50^ Upon endothelial apoptosis, pro-inflammatory effectors will be secreted for the recruitment of leucocytes recruitment to injured sites.^48, 50^ Herein, neutrophils are the first cells to adhere to endothelium and migrate to injured sites, where they eliminate dead cell debris for facilitating tissue repair.^41, 48, 49, 51^ However, the irradiated epithelium always leads to a reduced barrier function.^1^ On this occasion, neutrophils also participate in eliminating large amounts of pathogens within the enteric cavity through secreting high levels of anti-pathogenic effectors, such as myeloperoxidase (MPO);^52^ this process will increase the oxidative stress within injured intestine and reduce tissue regeneration thereby (Figure 2). Meanwhile, upon exposure of injured tissues to pathogens, the cytotoxic T lymphocytes will eliminate the cells infected by these foreign stimuli through secreting cytolytic substances, such as perforin and granzymes, and through Fas/Fas ligand binding-induced apoptosis (Figure 2).^53, 54^ Thus, the presence of pathogens promotes inflammation and induces a switch from an innate immune response to an adaptive immune response,^48, 54^ which has the inflammation extended in irradiated sites.

### Neuroimmune event: interactions between mast cells and enteric neurons

**Figure 3:**
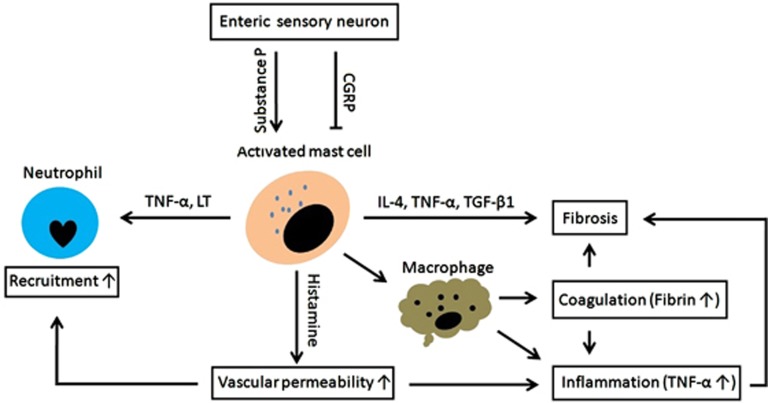
The contribution of neuroimmune interactions to RE development. Upon being irradiated, the mast cells will be activated, presenting the release of pro-inflammatory and pro-fibrotic effectors, including TNF-*α*, histamine, LT, IL-4 and TGF-*β*1. These actions will be amplified by substance P, whereas be reversed by CGRP. LT, leukotriene; CGRP, calcitonin gene-related peptide

The neuroimmune interactions between enteric neurons and mast cells formulate a network for controlling intestinal responses to ionizing irradiation, presenting the lesions of mucositis and fibrosis.^55^ But the mast cells distinguish their contributions to the pathogenesis of RE.^55^ Upon being irradiated, the mast cells within intestinal mucosa will be activated, presenting the release of some effectors, such as TNF-*α* and leukotriene (LT), for attracting neutrophils to clear bacteria-induced infection (Figure 3).^55^ Moreover, these activated mast cells will enhance TNF-*α* secretion by macrophages as well as macrophage-mediated fibrin deposition for coagulation (Figure 3).^55^ But for the mast cells in connective tissue, their contribution to RE is secreting some pro-fibrotic effectors, such as TGF-*β*1, IL-4 and TNF-*α* (Figure 3).^55^ Although the exact mechanism by which neuroimmune interactions regulate the development of RE remains unknown, it is clear that the mediators from enteric sensory neurons, such as substance P and calcitonin gene-related peptide (CGRP), are critical for regulating the activation of mast cells. Herein, substance P is capable of amplifying the pro-inflammatory and pro-fibrotic responses of mast cells to ionizing irradiation through enhancing the secretions of histamine, TNF-*α* and TGF-*β*1 by these cells, whereas the CGRP protects intestine against radiation-induced injuries (Figure 3).^55^ From this aspect, it is rational to propose that substance P is even important in deciding the severity of RE through activating mast cells.

### Microbial event: dysbiosis post-irradiation

McLaughlin *et al.*^56^ reported that the germ-free mice present radiation resistance comparing with conventional mice, indicating a potential link between the microbiota and host response to ionizing irradiation. As we are aware, the healthy gut contains nearly 300–500 bacterial species.^57^ These commensal bacteria confer the intestine a barrier function, presenting persistent epithelial turnover and vigorous immunity.^57^ For example, the NF-*κ*B signaling pathway in epithelial cells and some intestine-specific immune cells will be activated upon the interactions between their toll-like receptors and luminal bacteria, which stimulate the epithelial proliferation as well as lead to immune tolerance of intestine to foreign antigens.^58^ However, when the epithelium loses its integrity post-irradiation, the commensal bacteria will transmigrate into intestinal tissue, functioning as bioterrorists for even triggering sepsis.^57, 58^ In this context, dysbiosis commonly occurs. Manichanh *et al.*^59^ reported that the bacterial constitution of feces is altered after the patients receiving pelvic radiotherapy, presenting the increased clusters of Bacilli and Actinobacteria, whereas decreasing in clostridial cluster. Besides, previous data indicated that the microbiota confer the intestinal endothelium and lymphocyts radiation sensitivity, which is linked to the suppression of fasting-induced adipose factor by luminal microbiota.^60^ To a certain extent, this finding corresponds to that endothelial injuries are the primary lesions of RE.^40^ In current opinions, the interactions between the microbiota and host regulate the intestinal responses to ionizing irradiation, and not all intestinal bacteria promote pathogenesis of RE.^57, 58^ For example, the microbiota can induce epithelial expression of nucleotide-binding oligomerization domain-containing protein 2 (NOD-2) against ischemia/reperfusion-induced hypoxic stress and autophagy,^61^ indicating the bacteriotherapy for RE is also promising.^62^

### Fibrotic event: fibrogenesis after tissue injuries

Radiation-induced injuries in epithelium and endothelium have a central role in initiating intestinal fibrosis, which can be promoted by the extended inflammation within injured sites.^24, 25^ Upon epithelial injuries, irradiated intestine will reduce its barrier functions.^1^ On this occasion, the innate immune cells, such as macrophages and dendritic cells (DCs), will be activated by pathogens in enteric cavity, leading to secretion of pro-inflammatory cytokines, such as TNF-*α*, IL-1, IL-4 and IL-8.^63^ These cytokines activate some adaptive immune cells, such as Th2 and Th17 cell, which separately secrete IL-13 and IL-17 for promoting tissue remodeling (Figure 2).^41, 63^ Upon endothelial injuries, the blood vessels will increase their permeability, enabling recruitment of pro-inflammatory cells to injured sites.^41^ Among these infiltrated cells, the monocyte-macrophage system is reported to facilitate fibrosis through secreting PDGF, CTGF and TGF-*β*1, allowing for fibroblast-myofibroblast transition, myofibroblast proliferation and ECM deposition at injured sites (Figure 2).^64, 65^

## Stem Cell-Based Regenerative Therapy for Rodent Models of RE

In the past decade, extensive efforts have been made in stem-cell based therapy for rodent models of RE (Table 1).

### The therapeutic effects of MSCs on RE

To our knowledge, the first study was performed by Sémont *et al.*^66^ Their data showed that human MSCs exhibited potentials for maintaining the integrity of irradiated intestine.^66^ After MSC intervention, the irradiated mice survived longer than controls, and the epithelium showed hypertrophic villi, comprising increased numbers of proliferative cells and fewer apoptotic cells in the crypts. In addition, the newborn villus maintained its absorptive function via normal levels of Na^+^-K^+^-ATPase expression.^67^ Similarly, Kudo *et al.*^68^ reported that MSCs could extend the life span of irradiated mice, which was distinct from their previous data by using embryonic stem cells (ESCs) transplantation.^69^ Regarding the repair actions by MSCs, Saha *et al.*^70^ reported that MSCs could protect irradiated intestine by increasing serum levels of R-spondin1, KGF, IL-10 and PGE2, which function as effectors for promoting proliferation and inhibiting both apoptosis and inflammation within irradiated intestine. Besides, MSCs were capable of promoting epithelial regeneration using their secretion of IL-6.^71^ Moreover, the MSC-conditioned medium also exhibited the pro-regenerative potentials for irradiated epithelium.^72^ Besides, we found that MSC infusion could accelerate neovascularization within irradiated sites by triggering the intrinsic repair action of upregulated expressions of SDF-1, VEGF, basic FGF and Flk-1.^73^ Afterwards, by using a pig model of radiation proctitis, Linard *et al.*^74^ reported that repeated autologous transplantation of MSCs could counter act the inflammation in rectal mucosa by increasing host IL-10 production, and protect rectum against radiation-induced fibrosis by reducing local Col1a2/Col3a1 and TGF-*β*/CTGF expression and altering the matrix metalloproteinase (MMP)-tissue inhibitor of metalloproteinase (TIMP) balance as well. Similarly, based on using a rat model of radiation proctitis, Bessout *et al.*^75^ reported that autologous transplantation of MSCs was capable of mitigating the aberrant inflammation in the colorectal mucosa by elevating the local levels of glucocorticoid, which would inhibit the proliferation and induce apoptosis in radiation-activated T cells. These preclinical studies indicate that MSC-based therapy is potent for repairing the lesions associated with RE. Especially for using heterogenic MSCs, the host repair can be triggered as well, attributing to the autocrine/paracrine actions achieved by MSCs.

### The MSC-based gene therapy for RE

The chemotactic properties of MSCs enable themselves to be used as delivery vehicles for the targeted secretion of tissue repair factors at injured sites. Herein, several lines of evidence showed that radiation-induced upregulation in CXCL12 expression is important for the homing of MSCs to injured sites through the interaction between CXCR4 and CXCL12.^76, 77, 78^ For improving the homing efficacy of MSCs toward irradiated intestine, Zhang *et al.* ^79^ established the MSCs, overexpressing CXCR4 gene, and found that the restoration of epithelial integrity was accelerated upon intervention by CXCR4 gene-modified MSCs. In another study, Yang *et al.* ^80^ reported that manganese superoxide dismutase (MSD) gene-modified MSCs were more effective than control MSCs in reducing mortality of irradiated mice, relieving gastrointestinal symptoms and restoring epithelial integrity with a spot of apoptotic cells in irradiated mice. Moreover, Hu *et al.*^81^ revealed that MSCs overexpressing Trx-1 gene were powerful in reducing oxidative stress in the irradiated intestine. Overall, the superior effectiveness of gene-modified MSCs over unmodified MSCs in healing RE can be attributed to the dual therapeutic effects of the high expression of ectopic genes and the intrinsic functions of MSCs.

## Putative Actions Involved in RE Resolution by MSC Infusion

**Figure 4:**
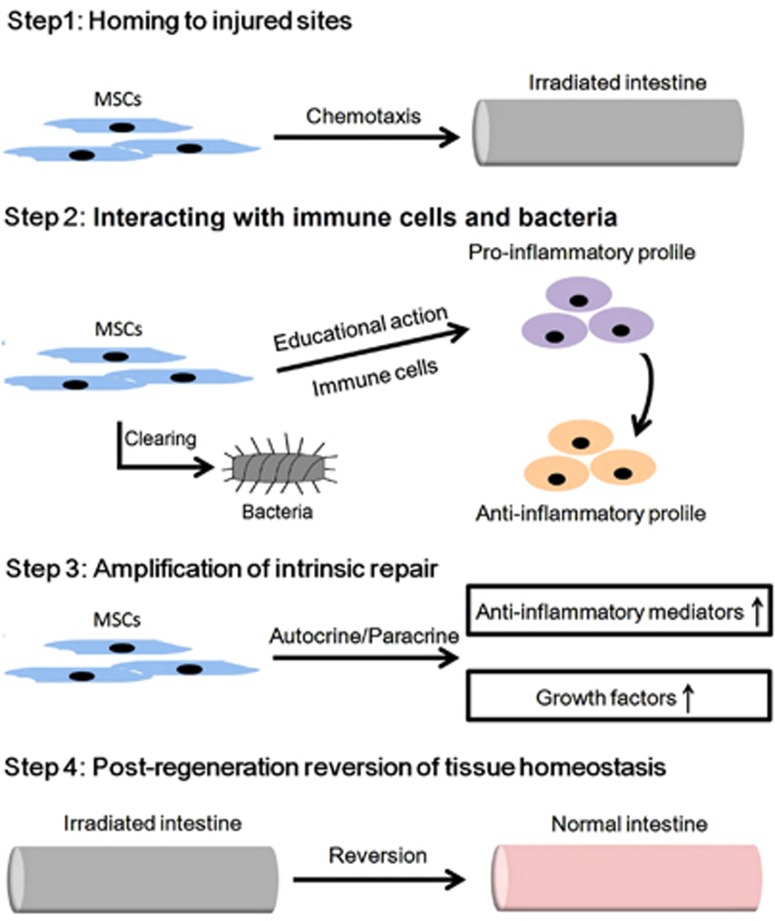
The putative actions by which MSCs repair radiation enteropathy. Four steps are involved in the processes of MSCs healing injuries in irradiated intestine, including cell-homing, interacting with immune cells, boosting intrinsic repair actions and reversing the homeostasis of injured tissue

At present, the mechanisms involved in the repair of irradiated intestine by MSCs are still not fully investigated. But according to recent advances, we suggest several putative actions of RE management achieved by MSCs. In our opinion, the putative actions by which MSCs repair RE can be summarized as follows (Figure 4). Primarily, the engrafted MSCs induce infiltrated immune cells to switch from pro-inflammatory to anti-inflammatory cytokine secretion, resulting in milieus that promote anti-inflammatory events. As a secondary effect, repair responses are boosted by systemic events, such as elevated levels of regenerative facilitators, despite the rapid disappearance of donor MSCs.^82^ Thus, benign cytokine milieus cause regeneration of the injured intestine to be accelerated.

### Step 1: homing to injured sites

Homing of infused MSCs to injured sites can be regarded as a prerequisite. According to recent data, several events mediated this homing process, such as CXCR1/2-CXCL8, CXCR4-CXCL12, CX_3_CR1-fractalkine, CCR7-CCL21 and ICAM-1/VCAM-1.^77, 83, 84, 85^ Upon these molecular bindings, MSCs first adhere to the endothelium, forming a defensive barrier against pathogens together with the pre-existing pericytes.^86^ The foreign MSCs then migrate to the laminar propria, where they perform pro-regenerative functions.^87^

### Step 2: interacting with immune cells and bacteria

**Figure 5:**
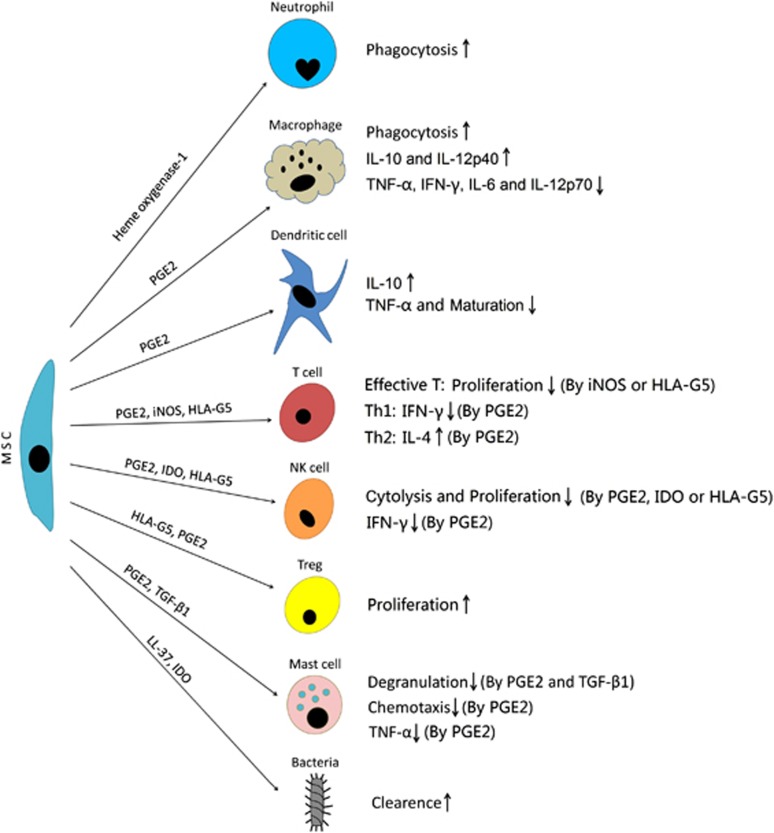
The interactions between MSCs and immune cells/Bacteria. Upon co-culturing with MSCs, the pro-inflammatory profiles of immune cells, including macrophages, dendritic cells, T effector cells, NK cells and mast cells, will be altered into the anti-inflammatory ones. By contrast, the proliferation of Tregs will be promoted by MSCs. Besides, MSCs have the anti-bacterial potentials, and the phagocytosis by neutrophils and macrophages will be strengthened by MSCs

Cytokines secreted by both immune cells and MSCs mediate crosstalk among these cell types through regulatory feedback mechanisms, termed as ‘Educational action' of MSCs (Figure 5).^17^ The engrafted MSCs will alter the inflammatory milieus through interacting with infiltrated immune cells via secretion central immune mediators, including IL-10, PGE2, iNOS, IDO and HLA-G5.^17^ For example, in a co-culturing system, BM-MSC increases the IL-10 secretion and decreases the TNF-*α* secretion in DCs; reduces IFN-*γ* secretion in Th1 cells and natural killer (NK) cells, and increases IL-4 secretion in Th2 cells.^88^ Under the same conditions, macrophages increase IL-10 and IL-12p40 secretion, while reducing TNF-*α*, IFN-*γ*, IL-6 and IL-12p70 secretion.^89^ These effects have also been observed *in vivo*: Németh *et al.* ^90^ found that MSC infusion-induced elevation of host PGE2 has a central role in enhancing IL-10 synthesis by monocytes and macrophages from the lungs of septic mice, while reducing IL-12 secretion from these immune cells. These events inhibited neutrophil infiltration and IL-12-induced activations of both NK cells and cytotoxic T lymphocytes.^90, 91^

In addition to the antagonistic effects of MSCs on immune cell pro-inflammatory profiles, the MSCs can inhibit the proliferation of effective T lymphocytes through secreting iNOS or HLA-G5, inhibiting DC maturation using PGE2, and inhibiting NK-mediated cytolysis and NK proliferation using PGE2, HLA-G5 or IDO (Figure 5).^92, 93, 94^ In contrast to their anti-proliferative effects on such immune cells, MSCs will stimulate proliferation of regulatory T cells (Tregs) via secreting HLA-G5 and PGE2 (Figure 5).^94, 95^ Recent *in vivo* data also confirmed that MSC infusion can increase the Treg number in mice with colitis; this increase is dependent on MSCs migrating to the spleen and interacting with splenic CD11b^+^ innate immune cells.^96, 97^ But for radiation proctitis, current opinion on whether IL-10 and/or Tregs participate in suppressing mucosal inflammation seems to be contradictive. A previous study found that the antagonistic effect of MSCs on the inflammation in colorectal mucosa of rat exhibited the IL-10/Treg-independent manner, which is opposite to the finding by using a pig model.^74, 75^ In spite of similar strategies in establishing animal models of radiation proctitis and carrying out autologous transplantation, the mechanism by which MSCs mitigate intestinal inflammation probably varies among species. In addition, the following issues, including delivery times and doses of MSCs, appear to affect the host responses in clearing systemic or local inflammation.

As described above, neuroimmune interactions and dysbiosis contribute to the pathogenesis of RE. Fortunately, recent data suggested that MSCs antagonized the activation of mast cells by secreting PGE2 and TGF-*β*1, leading to decreased degranulation, reduced ability of chemotaxis and reduced release of TNF-*α* by mast cells (Figure 5).^98, 99^ Moreover, accumulative evidence suggest that human MSCs are powerful in protecting against Gram-negative bacteria-induced sepsis, relying on their secretions of LL-37, IDO and heme oxygenase-1 (HO-1), and strengthening the phagocytosis by neutrophils and macrophages as well (Figure 5).^100, 101, 102, 103^

### Step 3: amplification of intrinsic repair

To date, it is still difficult to define the extent to which allogenic/heterogenic MSCs contribute to tissue regeneration, because immune rejection driven by recipient CD4^+^ and/or CD8^+^ T lymphocytes and oxidative stress within injured areas were reported to limit viability of infused MSCs.^82, 104^ But relatively few ectopic MSCs can lead to excellent therapeutic effects on injured host tissues that have a lost a large number of functional cells. A previous study reported that enhanced tissue repair achieved despite donor MSCs being rapidly eliminated after transplantation.^105^ From this point, we conclude that MSCs act as facilitators via triggering and boosting systemic repair responses. In spite of no consensus on whether the anti-inflammatory effect of MSCs on irradiated rectum of IL-10 and/or Treg involvment, reduced inflammation is always achieved after infusion of MSCs.^74, 75^ Combining with the actions of MSCs in clearing bacteria and minimizing the activation of mast cells, benign milieus for tissue regeneration are established, which will be beneficial to attract host bone marrow progenitors for reconstructing the niches of CBC stem cells and building on vasculature.^106, 107^ On this basis, by using the assistances from locally upregulated mitotic facilitators,^70, 83^ re-epithlialization and angiogenesis/neovascularization in the injured intestine are accelerated thereby.

### Step 4: post-regeneration reversion of tissue homeostasis

When the neo-formed epithelium or endothelium is restored to its normal size and structure, p53-mediated cell-cycle arrest halts cell proliferation and prevents tissue hypertrophy. Levels of cytokine or hormone secretion are altered in the host tissue to maintain physiological epithelial homeostasis.^108, 109^ This process provides an adequate blood supply to the gut, eliminates inflammation and prevents fibrosis from developing in the irradiated intestine.

## MSC and Cancer: A Latent Factor Affecting Security of MSCs for RE

The clinical responses of four Epinal patients to MSC-based therapy preliminarily revealed the specific roles of MSCs in treating pelvic radiotherapy-induced injuries in rectum and in bladder.^6, 23^ Although no evidence indicating the relapses in their prostate cancers after MSC intervention,^23^ the debate over whether MSCs promote the growth and metastasis of cancer cells or initiate new cancers persists for a long time, because truth lies in that majority of RE patients are still cancer survivors.^110^

### ‘Negative effects' of MSCs on tumor growth and their oncogenicity

Unrestricted cancer cell proliferation is largely driven by dysregulated growth signals originating from mutated genes.^110^ Some researchers believe that MSC-initiated immune suppression, immune cell dysfunction, cell division and angiogenesis could promote cancer progression.^111, 112^ In addition, MSCs would undergo spontaneous malignant transformation during a culture period of 105 weeks.^113, 114, 115^ Genomic instability, such as spontaneous p53 mutation in aged or in p21-deficient MSCs, was related to fibrosarcoma formation *in vivo*.^114, 115, 116, 117^ By contrast, the risk of genetic mutations or cell aging could be minimized, if MSCs are cultured for less than 16 weeks.^118, 119^

### ‘Positive effects' of MSCs on tumor growth and their anticancer potential

Recent data also suggested that MSCs exhibited potentials for inhibiting tumor growth through arresting cell cycling and activating signaling pathways related to cell death (Table 2).^120, 121, 122, 123, 124, 125, 126, 127, 128, 129, 130, 131, 132, 133^ For example, human adipose MSCs could inhibit tumor cell expansion via secreting Dickkopf-related protein 1, a Wnt signaling antagonist.^134, 135, 136^ Another anticancer activity of MSCs involved the blockade of PI3K/Akt signaling pathway, leading to increased levels of cell-death related molecules, while decreased levels of cell survival-related molecules (Table 2).^121, 133, 137^

In addition, MSC-based gene therapy for various solid tumors improves host anticancer responses through releasing the foreign gene-encoded proteins, such as iNOS, IL-2 and IL-12.^138, 139, 140^ Optimistically, some clinical trials for evaluating the specific potentials of MSCs on treating cancer and cancer regimen-related disorders are being carried out (*ClinicalTrials.gov* data). Relevant results will be referred for anticipating the potential risks related to MSC-based therapy.

## Future Efforts into MSC-Based Therapy for RE Patients

Because of the success in treating Epinal patients by using MSCs, a new protocol for treating late severe damages of abdominal radiotherapy has been performed in Epinal Medical Center since 2013.^141^ Certainly, the ‘Epinal experiences' will deserve being referred worldwide in the future. If so, then MSC-based therapy is merely an attractive tool in managing RE patients. And clinical use of MSCs should focus on the following rules: (i) Ensuring the quality control of MSCs, including the processes for generating MSCs, identifying MSCs, detecting pathogens and endotoxin, and removing residual supplements.^142^ A detailed protocol for generating clinical grade human MSCs can be consulted,^143^ if using autologous MSCs. If using allogenic MSCs, then some FDA-approved products are available nowadays. But when allogenic MSCs are poorly tolerated, MSC exosomes are expected to become alternative candidates for managing RE. First, MSC exosomes show the potentials in mediating tissue regeneration because they contain a broad spectrum of bioactive substance.^144^ For example, the adenylate kinase and nucleoside-diphosphate kinase within exosomes are critical in increasing ATP production within injured cells.^144^ Additionally, CD73 molecules presenting in MSC exosomes are capable of hydrolyzing AMP into adenosine, an activator for cell survival by stimulating MAPK and PI3K/Akt signaling pathways.^144^ Moreover, MSC exosomes contain more than 200 immunomodulatory proteins, which confer the anti-inflammatory capability on exosomes.^144^ Second, the MSC exosomes seem to be stable *in vitro,* because their integrities and sizes were seldom affected by repeated freezing-thawing,^145^ allowing single extraction of exosomes for multiple administrations clinically. (ii) Formulating a protocol for MSC therapy for RE, including the delivery route of MSCs, timing and frequency of injection, the optimal dose and passage.^146^ (iii) Making countermeasure against possible complications or emergencies related to MSC infusion, such as fever, allergy or even shock.^142, 147^ (iv) Establishing the criteria for evaluating the effectiveness achieved by MSC infusion in RE resolution, which somewhat guarantees the researchers avoid overstating the positive outcomes.^148, 149, 150^

## Conclusions

Overall, data from preclinical study and Epinal case report highlight the essentiality of futuristic use of MSCs in RE management. MSC-based therapy is expected to have beneficial effects on the quality of life of RE patients.

## Figures and Tables

**Table 1:** Advances in regenerative therapy for rodent models of RE

**Year**	**Researcher (Ref.)**	**MSC/ESC (gene-modified), source and specie**	**Animal**	**Total dose (position)**	**Main findings**
2006	Sémont *et al.*^66^	BM-MSC, human	NOD/SCID mice	3.5 Gy (WBI)+4.5 Gy (AI)	•Hypertrophic villus-crypt axis •Maintaining the epithelial integrity
2007	Kudo *et al.*^69^	ESC, 129/Sv cell line	ICR *nu/nu* mice	30 Gy (AI)	•ESCs are committed to epithelial cells •Fail in prolong the lifespan of irradiated mice
2008	Zhang *et al.*^79^	BM-MSC (CXCR4), *β-Gal*-transgenic mice	C57BL/6 J mice	13 Gy (AI)	•Hypertrophic villus-crypt axis •Reducing intestinal permeability
2010	Kudo *et al.*^68^	BM-MSC, C57BL/6	ICR *nu/nu* mice	30 Gy (AI)	•Decreasing mortality rate •Increasing body weight •Maintaining epithelial integrity
2010	Sémont *et al.*^67^	BM-MSC, human	NOD/SCID mice	3.5 Gy (WBI)+7 Gy (AI)	•Decreasing mortality rate •Maintaining absorptive function of epithelium •Restoring the integrity of epithelium •Increased number of proliferative cells in crypt •Decreased number of apoptotic cells in crypt
2011	Saha *et al.*^70^	BM-MSC, C57BL/6 mice	Dipeptidlyl-peptidase-deficient mice; Lgr5-EGFP-IRES-CreERT2 mice	10.4 Gy (WBI) 18 Gy (AI)	•Decreasing motality •Maintaining the integrity of epithelium •Mitigating inflammation
2012	Francois *et al.*^71^	BM-MSC, C57BL/6 and IL-6^−/−^ (B6. 129S2-Il6^tmlKopf^/J)	Barb/C mice	9 Gy (WBI)	•Protecting mice against radiation-induced death •Hypertrophic villi •Stimulating epithelial regeneration mainly by MSC-derived IL-6
2012	Gao *et al.* ^72^	UC-MSC,^a^ human	Barb/C mice	7, 8.5, 10, 11.5 and 13 Gy (AI)	•Extending the life span of mice receiving 10 Gy •Hypertrophic villi •Maintaining epithelial integrity
2013	Chang *et al.*^73^	Ad-MSC, human	Sprague-Dawley rats	15 Gy (AI)	•Decreasing mortality rate •Increasing body weight •Mitigating inflammation •Accelerating neovascularization •Restoring epithelial integrity
2013	Linard *et al.*^74^	BM-MSC, Göttingen pig	Göttingen pigs	21–29 Gy (PI)	•Mitigating inflammation •Inhibiting fibrosis in irradiated site •Facilitating angiogenesis in irradiated site
2013	Yang *et al.*^80^	BM-MSC (MSD), human	NOD/SCID mice	4–6 Gy (AI)	•Decreasing mortality rate •Reducing the number of apoptotic cells •Mitigating inflammation •Maintaining integrity of epithelium
2013	Hu *et al.*^81^	UC-MSC (Trx-1), human	NOD/SCID mice	4.5 Gy (WBI)	•Maintaining epithelial integrity •Reducing oxidative stress
2014	Bessout *et al.*^75^	BM-MSC, Sprague-Dawley rats	Sprague-Dawley rats	Gy (PI)	•Reducing mucosal inflammation •Promoting the proliferation of epithelial cells •Inducing apoptosis of radiation-activated T cells •Inhibiting infiltration and proliferation of T cells •Elevating the local levels of corticosterone •Upregulating the local expression of HSD11b1-steroidogenic enzyme

Abbreviations: BM, bone marrow; Ad, adipose tissue; UC, umbilical cord; WBI, whole body irradiation; AI, abdominal irradiation; PI, Pelvic irradiation; HSD11b1, 11*β*-hydroxysteroid dehydrogenase type 1.

a Using conditioned medium of UC-MSCs.

**Table 2:** Typical cases indicating the antagonistic effects of MSCs on tumor growth

**Researcher (Ref.)**	**Study type**	**Tumor/cell line**	**MSC source/species**	**Gene modification (yes/no)**	**Molecular alteration in tumor cells**	**Main findings**
Khakoo *et al.*^121^	*Ex/In vivo*	Kaposi's sarcoma (KS)	BM human	No	Akt activity↓	•Reducing KS cell growth *ex vivo* •Inhibiting KS tumorigenesis *in vivo*
Li *et al.*^122^	*Ex/In vivo*	Multiple myeloma (MM)/H929	Placenta human	No	Unknown	•Dose-dependent inhibition of MSCs on MM growth *in vivo* •Inducing apoptosis of osteoc-last precursor *in vivo* •Promoting bone formation *in vivo* •Inhibiting growth of H929 cell *ex vivo*
						
Ahn *et al.*^123^	*Ex/In vivo*	Melanoma/AS375SM and A375P	Ad human	No	G0/G1 arrest	•Inducing the apoptosis in AS375SM and A375P cells *ex vivo* •Inhibiting melanoma growth *in vivo*
Nasuno *et al.*^124^	*Ex/In vivo*	Azoxymethane-induced colonic carcinoma/IEC-6	BM rat	No	G1 arrest pSmad2, I*κ*B*α* and p21↑ 79 genes of WNT signaling pathway↓	•Reducing tumor number *in vivo* •Altering the WNT and TGF-*β*/Smad signaling pathways in tumorigenesis *in vivo* •Suppressing AARGC(a genotoxic carcinogen)-induced acute apoptotic response *in vivo* •Reducing the formation of aberrant crypt foci *in vivo* •Reducing the number of DNA adducts of O6MeG in colonic epithelial cells •Inducing apoptosis of IEC-6 cells *ex vivo*
Katsuno *et al.*^125^	*Ex/In vivo*	1,2-dimethylhydrazine and dextran sulfate sodium-induced colorectal tumor/ACL15	BM rat	No	TGF-*β*1↑	•Reducing the number of aberrant crypt foci *in vivo* •Inhibiting the proliferation of ACL15 cells *ex vivo*
Lu *et al.*^126^	*Ex/In vivo*	Hepatoma/H22 Lymphoma/YAC-1 and EL-4 Insulinoma/INS-1	BM mouse	No	p21 and Caspase-3↑ G0/G1 arrest	•Dose-dependent inhibition of MSCs on tumor cell growth *ex vivo* •Inhibiting hepatoma growth *in vivo*
Qiao *et al.*^127^	*Ex/In vivo*	Hepatoma/H7402 and HepG2	DT human	No	*β*-Catenin, Bcl-2, PCNA and survivin↓	•Inhibiting hepatoma growth *in vivo* •Inducing apoptosis of H7402 cells *ex vivo* •Reducing proliferation of H7402 cells *ex vivo* •Altering the malignant phenotype of HepG2 cells *ex vivo*
Abd-Allah *et al.*^128^	*Ex/In vivo*	Hepatoma/Hepa 1-6	BM mouse	No	Caspase-3, p21 and p53↑ Bcl-2 and survivin↓	•Inhibiting growth of Hepa 1–6 *ex vivo* •Reducing serum alanine transaminase (ALT), aspartate transaminase (AST) and albumin levels *in vivo* •Inducing dysplasia of hematoma *in vivo*
Abdel Aziz *et al.*^132^	*In vivo*	Experimental hepatocellular carcinoma	BM rat	No	*β*-Catenin, PCNA, cyclin D and survivin↓	•Reducing liver damage •Decreasing the serum ALT, AST and *α*-fetoprotein levels
Ahn *et al.*^129^	*Ex/In vivo*	T-cell lymphoma/EL4	Ad human	No	G0/G1 arrest	•Inducing apoptosis of EL4 cells *ex vivo* •Inhibiting lymphoma growth *in vivo* •Prolonging survival time of lymphoma bearing mice
Chien *et al.*^130^	*In vivo*	Glioma/U87MG	BM human	No	Unknown	•Limiting the progression of glioma
Vegh *et al.*^131^	*In vivo*	Mammary tumor	Placenta human	No	Unknown	•Inhibiting the growth of primary mammary tumor •Inhibiting the development of new tumors
Ma *et al.*^133^	*Ex/In vivo*	Mammary tumor/MDA-MB-231 and MCF-7	UC human	No	PI3K/Akt↓ G2/M arrest	•Decreasing proliferation of MDA-MB-231 and MCF-7 cells *ex vivo* •Inducing apoptosis of MDA-MB-231 and MCF-7 cells *ex vivo* •Inhibiting the growth of mammary tumor *in vivo*
Zhu *et al.*^134^	*Ex vivo*	Leukemia/K562 and HL60 and Mammary tumor/MCF-7	Ad/Human	No	G0/G1 arrest; *β*-catenin, c-Myc and Cyclin D2↓ p21CIP1 and p27KIP1↑	•Inhibiting proliferation of tumor cells
Han *et al.*^137^	*Ex/In vivo*	Prostate cancer/PC-3	UC human	No	Cleaved caspase 3/9, PARP, JNK and Bax↑ PI3K/Akt, ERK↓Bcl-2, Bcl-xl, survivin Mcl-1 and clAP -1↓	•Inducing apoptosis of PC-3 cells *ex vivo* •Inducing PC-3 cell-death *in vivo*
Xiang *et al.*^138^	*Ex/In vivo*	Fibrosarcoma/Rif-1	BM rat	Yes (iNOS)	Unknown	•Inhibiting Rif-1 tumor growth *in vivo* •Inducing apoptosis of Rif-1 cells *ex vivo*
Nakamura *et al.*^139^	*Ex/In vivo*	Glioma/9 L	BM rat	Yes (IL-2)	Unknown	•Inhibiting proliferation of 9 L cells *ex vivo* •Inhibiting the growth of glioma *in vivo* •Prolonging the survival time of glioma bearing mice *in vivo*
Gao *et al.*^140^	*In vivo*	Renal cell carcinoma (RCC) /786-0	BM human	Yes (IL-12)	Unknown	•Reducing the growth of 786-0 RCC *ex vivo* •Prolonging the survival time of RCC bearing mice *in vivo*

Abbreviations: Ad, adipose; BM, bone marrow; UC, umbilical cord; DT, dermal tissue.

## Abbreviations

CBC: crypt base columnar
CGRP: calcitonin gene-related peptide
DC: dendritic cell
DSB: double-strand break
ECM: extracellular matrix
FI: fecal inconvenience
GVHD: graft *versus* host disease
HO-1: heme oxygenase-1
IBD: inflammatory bowel disease
LT: leukotriene
RE: radiation enteropathy
SLE: systemic lupus erythematosus
TLR: toll-like receptor
MMP: matrix metalloproteinase
MPO: myeloperoxidase
MSC: mesenchymal stem cell
MSD: manganese superoxide dismutase
NE: neutropenic enterocolitis
NF-*κ*B: nuclear factor-kappa B
NOD-2: nucleotide-binding oligomerization domain-containing protein 2
OAR: organ at risk
TIMP: tissue inhibitor of metalloproteinase
Treg: regulatory T cell
vWF: von Willebrand factor
